# Intrinsic Disorder and Posttranslational Modifications: The Darker Side of the Biological Dark Matter

**DOI:** 10.3389/fgene.2018.00158

**Published:** 2018-05-04

**Authors:** April L. Darling, Vladimir N. Uversky

**Affiliations:** ^1^Department of Molecular Medicine, USF Health Byrd Alzheimer’s Institute, Morsani College of Medicine, University of South Florida, Tampa, FL, United States; ^2^Laboratory of New Methods in Biology, Institute for Biological Instrumentation, Russian Academy of Sciences, Pushchino, Russia

**Keywords:** intrinsically disordered proteins, intrinsically disordered protein regions, posstranslational modifications, multifunctional proteins, protein–protein interaction (PPI)

## Abstract

Intrinsically disordered proteins (IDPs) and intrinsically disordered protein regions (IDPRs) are functional proteins and domains that devoid stable secondary and/or tertiary structure. IDPs/IDPRs are abundantly present in various proteomes, where they are involved in regulation, signaling, and control, thereby serving as crucial regulators of various cellular processes. Various mechanisms are utilized to tightly regulate and modulate biological functions, structural properties, cellular levels, and localization of these important controllers. Among these regulatory mechanisms are precisely controlled degradation and different posttranslational modifications (PTMs). Many normal cellular processes are associated with the presence of the right amounts of precisely activated IDPs at right places and in right time. However, wrecked regulation of IDPs/IDPRs might be associated with various human maladies, ranging from cancer and neurodegeneration to cardiovascular disease and diabetes. Pathogenic transformations of IDPs/IDPRs are often triggered by altered PTMs. This review considers some of the aspects of IDPs/IDPRs and their normal and aberrant regulation by PTMs.

## Introduction

Protein posttranslational modifications (PTMs) represent an important means for the natural increase in the proteome complexity and serves as one of the factors (in addition to the allelic variations and alternative splicing, together with some other pre-translational mechanisms, e.g., mRNA editing, allowing production of numerous mRNA variants), defining the ability of one gene to efficiently encode a set of distinct protein molecules, known as proteoforms (Smith et al., 2013). Since in eukaryotic organisms, the number of functionally different proteins dramatically exceeds the number of protein-encoding genes, one can reasonably argue that the complexity of a biological system is mostly determined by its proteome size and not by the size of genome (Schluter et al., 2009). Although the number of human protein-coding genes is approaching 20,700 (ENCODE Project Consortium, 2012), the actual human proteome includes a few hundred thousand, if not millions, of functionally different proteins (Uhlen et al., 2005; Farrah et al., 2013, 2014; Kim et al., 2014; Reddy et al., 2015). Because PTMs can affect protein activity, folding, interactions, localization, stability, and turnover, they play an important role in defining the protein structure-function continuum, according to which a protein has multiple structurally and functionally different states, proteoforms, generated by various mechanisms (including PTMs) (Uversky, 2015, 2016a,b,c). It was also pointed out that combination of alternative splicing, PTMs, and intrinsic disorder represents an important means for promotion of the alternative, context-dependent states of gene regulatory networks, thereby serving as a critical tool for controlling of a broad range of cellular responses, including cell fate specification (Niklas et al., 2015).

Intrinsic disorder and protein conformational dynamics represent another means used by nature to generate proteoforms (Uversky, 2015, 2016a,b,c). In fact, it was pointed out that even without alternative splicing, PTMs, or mutations, any protein, being a dynamic conformational ensemble, represents a set of basic (or intrinsic, or conformational) proteoforms; i.e., molecules with identical amino acid sequences but with different structures and, potentially, with different functions. Obviously, any mutated, modified, or alternatively spliced form of a protein [i.e., any member of the inducible (or modified) proteoforms] also exists as a structural ensemble and thereby represents a set of conformational proteoforms (Uversky, 2015, 2016a,b,c). Protein functions and structural ensembles of basic and induced proteoforms can be significantly altered by placing proteins in crowded cellular environment. This environment originates from high concentrations of various biological macromolecules inside the cells (Zimmerman and Trach, 1991; van den Berg et al., 1999; Rivas et al., 2004) that limit available volume (Ellis and Minton, 2003) and restrict amounts of free water (Fulton, 1982; Zimmerman and Trach, 1991; Zimmerman and Minton, 1993; Minton, 1997; Minton, 2000; Ellis, 2001). Finally, functionality *per se* can be considered as a factor generating functioning proteoforms (Uversky, 2015, 2016a,b,c). In other words, because of all these factors, any given protein exists as a set of basic, induced and functioning proteoforms. As a result, the actual relationships between a gene and a protein function follow the ‘one-gene – many-proteins – many-functions’ concept (Uversky, 2015, 2016a,b,c). This is in a striking contrast to the classical ‘one-gene – one-protein’ view, where each gene produces a single enzyme for controlling a single step of a metabolic pathway (Beadle and Ephrussi, 1936; Beadle and Tatum, 1941; Horowitz et al., 1945; Horowitz, 1995).

Posttranslational modifications are included into the ‘dark matter of biology’ concept that is attributed to “important, yet invisible species of molecules and proteins that interact weakly but couple together to have huge and important effects in many biological processes… [and that] remains mostly hidden, because our tools were developed to investigate strongly interacting species and folded proteins” (Ross, 2016). Curiously, many PTMs are intimately linked to another component of the ‘dark matter of biology,’ namely intrinsically disordered proteins (IDPs) and IDP regions (IDPRs), making such disorder-centered PTMs the darker side of the biological dark matter. This review is dedicated to the analysis of an intriguing connection between intrinsic disorder and PTMs, thereby representing an attempt to shed some light to the darker corner of the dark matter of biology.

## What Is Intrinsic Disorder?

### Intrinsically Disordered Proteins, Their Abundance and Biological Functions

The protein functionality is not always associated with the presence of unique 3D structure in a protein molecule. Instead, functional states of many proteins constitute dynamic conformational ensembles of interconverting structures, where a whole protein or its noticeable regions lack stable tertiary and/or secondary structure (Wright and Dyson, 1999; Uversky et al., 2000; Dunker et al., 2001; Tompa, 2002; Uversky and Dunker, 2013). These floppy but biologically active proteins and regions are currently known as IDPs and IDPRs. In relation to the subject of this special issue, IDPRs frequently contain sites of proteolytic attack and include various PTM sites (Dunker et al., 2001; Iakoucheva et al., 2004; Radivojac et al., 2010; Pejaver et al., 2014).

**FIGURE 1:**
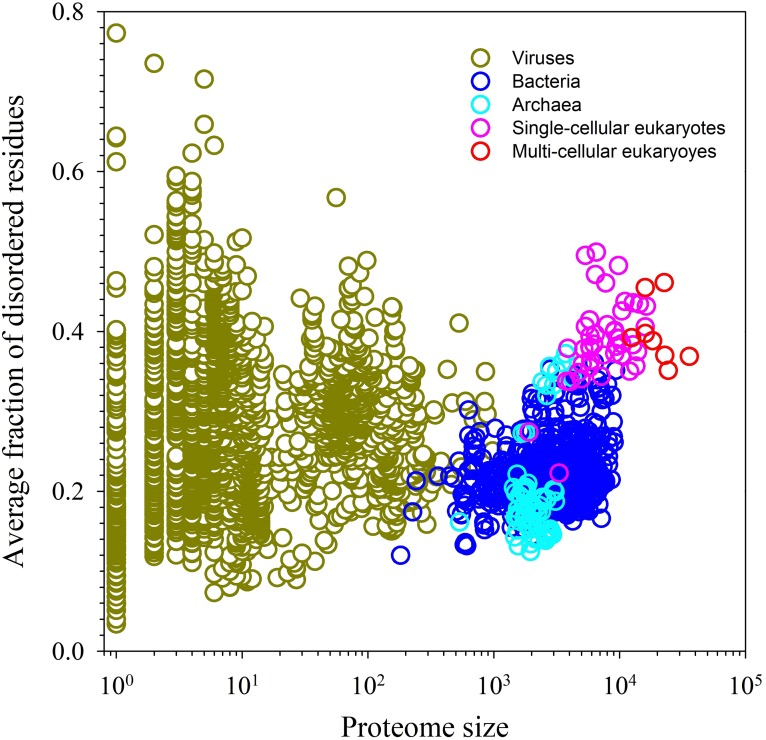
Abundance of intrinsic disorder in various proteomes.

**FIGURE 2:**
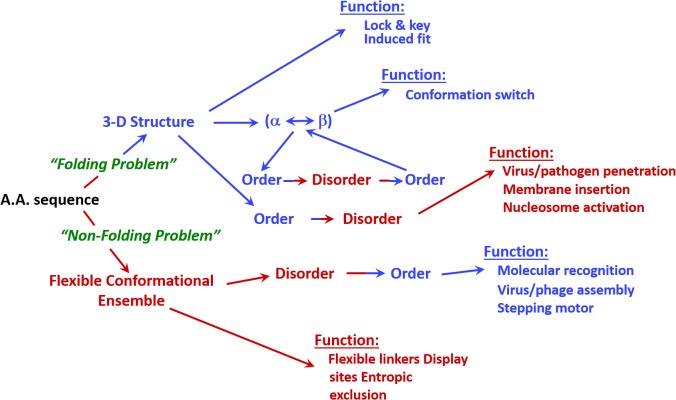
Involvement of intrinsic disorder in protein function. Note that the classical structure-function paradigm cannot describe many of the function proteins perform.

**Figure 1** shows that IDPs and IDPRs are abundantly present in all proteomes analyzed to date, with the proteome disorder levels typically increasing with the increase of the organism complexity (Romero et al., 1998; Dunker et al., 2000, 2001; Ward et al., 2004; Uversky, 2010; Xue et al., 2012; Peng et al., 2014). Furthermore, many IDPs/IDPRs are evolutionary conserved, indicating that at least some protein functionality is determined by intrinsic disorder. In fact, order and disorder are both needed for protein functionality, and functional repertoire ascribed to IDPs/IDPRs complement catalytic and transport functions of ordered proteins and domains (Radivojac et al., 2007). Therefore, the unbiased consideration of protein functionality should include both ordered proteins/domains and IDPs/IDPRs (Dunker et al., 2001, 2002a). This idea is illustrated by **Figure 2** showing that protein structure-function interrelationship includes two pathways, where the ‘sequence-to-structure-to-function’ paradigm can be utilized for the description of functionality of transport proteins and enzymes, whereas the ‘sequence-to-dynamic conformational ensemble-to-function’ paradigm represents a key for understanding the functions of IDPs/IDPRs preferentially involved in control, recognition, regulation, and signaling (Dunker et al., 2001, 2002a,b; Uversky, 2002a,b). Furthermore, the ‘protein trinity’ (Dunker and Obradovic, 2001) or the ‘protein quartet’ (Uversky, 2002a) models were used for the conceptualization of protein functionality. In these models, function of a protein can be associated with ordered, molten globule-like (collapsed-disordered), pre-molten globule-like (partially collapsed-disordered) or coil-like (extended-disordered) conformations and/or from the transitions between all these conformations (Dunker and Obradovic, 2001; Uversky, 2002a).

Some illustrative biological activities of IDPs/IDPRs include protein and RNA chaperone action, control of transcription and translation, storage of small molecules, location of PTM sites, regulation of the cell cycle and division, control of development, regulation and modulation of the self-assembly of large multi-protein complexes, and moderation of signal transduction, to name a few (Wright and Dyson, 1999; Uversky et al., 2000, 2005; Dunker and Obradovic, 2001; Dunker et al., 2002a,b, 2005, 2008a,b; Iakoucheva et al., 2002; Tompa, 2002, 2005; Uversky, 2002a,b, 2010; Dyson and Wright, 2005; Radivojac et al., 2007; Vucetic et al., 2007; Xie et al., 2007a,b; Cortese et al., 2008; Dunker and Uversky, 2008). The existence of at least 28 separate disorder-based functions was proposed based on the careful analysis of literature on >150 completely disordered proteins or proteins containing functional IDPRs (Dunker et al., 2002a). Globally, functional IDPs/IDPRs have very different modes of action serving as entropic chains, display sites, assemblers, effectors, chaperones, and scavengers (Tompa, 2002; Tompa and Csermely, 2004).

Because of their flexible nature, IDPs/IDPRs have some remarkable functional advantages over their ordered counterparts (Schulz, 1979; Pontius, 1993; Plaxco and Gross, 1997; Wright and Dyson, 1999; Dunker et al., 2001, 2002a, 2005; Dyson and Wright, 2002; Iakoucheva et al., 2002; Dyson and Wright, 2005; Uversky et al., 2005), including their ability to be promiscuous binders engaged in efficient interactions with different and often unrelated targets (Wright and Dyson, 1999; Uversky et al., 2000; Dunker et al., 2001; Uversky and Dunker, 2010). One of the said functional advantages of IDPs/IDPRs is their ability to (partially) fold (or undergo disorder-to-order transitions) upon interaction with specific partners (Schulz, 1979; Pontius, 1993; Spolar and Record, 1994; Plaxco and Gross, 1997; Wright and Dyson, 1999; Dunker et al., 2001, 2002a, 2005; Dyson and Wright, 2002, 2005; Iakoucheva et al., 2002; Oldfield et al., 2005; Uversky et al., 2005; Mohan et al., 2006; Cheng et al., 2007; Vacic et al., 2007; Tompa et al., 2009). In relation to the reversible signaling interactions, such binding-induced disorder-to-order transitions allow IDPs/IDPRs to participate in highly specific but weak interactions (Schulz, 1979; Dunker et al., 2001). Furthermore, IDPs/IDPRs can fold on a template-dependent manner. This provides them with an ability to attain very different structures at interaction with different binding partners (Kriwacki et al., 1996; Dunker et al., 2001; Oldfield et al., 2008; Hsu et al., 2012, 2013).

Although promiscuous interactivity and ability to fold at binding to specific partners represent a characteristic signature of disorder-based functionality, one should remember that IDPs/IDPRs have numerous ‘entropic chain activities.’ These activities rely on the flexibility, plasticity, and pliability of the protein backbone and therefore that do not require coupled binding and folding. The voltage gated ion channel can serve as an illustrative example of such entropic chain activities. Activity of this channel involves cyclic transitions between closed (sensitive to voltage), open, and inactive (insensitive to voltage) states (Armstrong and Bezanilla, 1977; Antz et al., 1997). In structure of such channels, a highly flexible ‘chain’ connects a small folded domain, ‘ball,’ to the channel opening. Channels utilize an entropic clock mechanism for inactivation, where the random motions of the ‘ball-and-chain’ unit provide a means for the ‘ball’ to eventually plug into the open channel and inactivate it (Armstrong and Bezanilla, 1977; Antz et al., 1997). It was pointed out that timing of such channels relies on flexibility and length of their disordered ‘chains.’ In fact, since the time of closure of such channels is inversely proportional to the square of the length of their ‘chains’ (Liebovitch et al., 1992), inactivation is faster when the ‘chain’ is shorter (Hoshi et al., 1990).

Entropic bristle domain (EBD); i.e., the time-averaged three-dimensional area swept out by the thermally driven motion of a disordered polypeptide, represents another example of entropic activity. Obviously, EBD that occupies a defined area but does not have a fixed structure is different from a traditional protein domain, which is a conserved part of a protein structure that can evolve, function, and exist independently of the rest of the polypeptide chain. Illustrative carriers of functional EBDs are given by the neurofilament proteins found in the major cytoskeletal components of the axonal cell, neurofilaments, that among other functions, are needed to maintain the bore of the axon (Brown and Hoh, 1997). The needed spacing between the neurofilaments is kept by the action of the entropic brush formed by EBDs that project from the NF-M and NF-H neurofilament proteins (Brown and Hoh, 1997).

Overall, multiple functional advantages were ascribed to IDPs/IDPRs to explain their prevalence in various proteomes and their broad use in various biological processes (Dunker et al., 1997, 1998, 2001; Wright and Dyson, 1999; Romero et al., 2001; Brown et al., 2002; Cortese et al., 2008). Natural abundance of IDPs/IDPRs is clearly related to their multifunctionality, which, in its turn, is linked to their specific structural features. Sections below consider peculiarities of structural organization and conformational behavior of IDPs/IDPRs to better understand the uniqueness of these intriguing members of protein universe.

### Some Basic Structural Properties of IDPs/IDPRs

**FIGURE 3:**
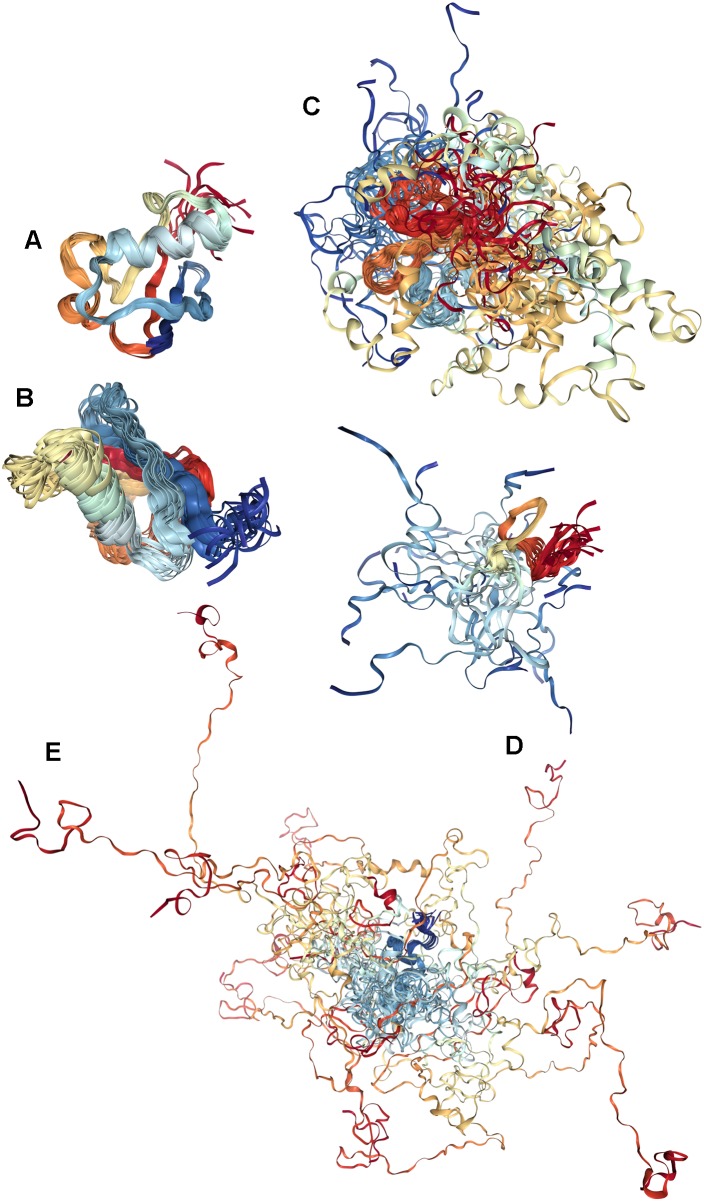
Illustrative examples of proteins with different levels of global disorder. **(A)** Ordered protein: NMR solution structure of bovine ubiquitin (PDB ID: 1V81) (Kitahara et al., 2005). **(B)** Protein with the collapsed (molten globule-like) disorder: NMR solution structure of Ubiquitin-like domain of NFATC2IP (PDB ID: 2JXX). **(C)** Protein with semi-extended (pre-molten globule-like) disorder: NMR solution structure of the domain II from the blood-stage malarial protein, apical membrane antigen 1 (PDB ID: 1YXE) (Feng et al., 2005). **(D)** Small protein with extended (coil-like) disorder: sunflower trypsin inhibitor 1 (SFTI-1) (PDB ID: 2AB9) (Mulvenna et al., 2005). **(E)** Large protein with extended (coil-like) disorder: NMR solution structure of Air2p protein, which is the key component of the Trf4/5p-Air1/2p-Mtr4p polyadenylation complex (TRAMP) (PDB ID: 2LLI) (Holub et al., 2012).

Structural description of IDPs/IDPRs relies on the dynamic conformational ensemble representation, where the members of such conformational ensembles interconvert on a number of timescales. By analogy with the conformational states of typical globular proteins that, depending on the environmental conditions, may exist in at least four different conformations: folded (ordered), molten globule, pre-molten globule, and coil-like (Uversky and Ptitsyn, 1994, 1996; Fink, 1995; Ptitsyn, 1995; Ptitsyn et al., 1995; Uversky, 2003; Turoverov et al., 2010), IDPs, being considered at the whole protein level, can be classified as native molten globules containing collapsed form of disorder, native pre-molten globules possessing semi-collapsed form disorder, and native coils with extended form of (Dunker et al., 2001; Uversky, 2003). This is illustrated by **Figure 3** schematically representing these three types of disorder at the whole protein level.

One should keep in mind, however, this classification represents a clear oversimplification, since order and disorder are not homogeneously distributed within a protein molecule. In fact, analysis of crystal structures of various proteins deposited in PDB revealed that they are not only characterized by the presence of regions with high B-factor that reflects the high uncertainty in atom positions in the model, but systematically contain long regions of missing electron density that often correspond to IDPRs (Radivojac et al., 2004) [in fact, only about 7% of PDB entries devoid intrinsic disorder (Le Gall et al., 2007)], and also contain ambiguous regions, where different structures of the same protein disagree in terms of the presence or absence of missing residues (Le Gall et al., 2007; DeForte and Uversky, 2016). It is likely that similar levels of disorderedness (collapsed, semi-collapsed, and extended forms of disorder) discussed for the whole proteins can also be found in protein regions, indicating that both IDPs and IDPRs can be differently disordered. These observations suggest that proteins, in general, are characterized by non-homogeneous distribution of foldability and, as a consequence, by a high spatiotemporal heterogeneity, where different parts of a protein molecule are ordered (or disordered) to a different degree (Uversky, 2013a,c). In other words, globally, structural space of functional proteins represents a continuous spectrum of conformations with different degree and depth of disorder. This structural spectrum spreads from fully ordered to completely disordered proteins and includes a myriad of differently (dis)ordered species between these two extremes (Uversky, 2013a,c). Therefore, protein structural space represents a structure-disorder continuum with no clear boundary between ordered proteins and IDPs (Uversky, 2013c). It was also pointed out that because of the presence of different levels and depths of intrinsic disorder, proteins are characterized by the mosaic structure and typically contain foldons (i.e., independently foldable regions), inducible foldons (disordered regions that can fold at interaction with binding partner), semi-foldons (IDPRs that are always in the semi-folded state), non-foldons (IDPRs with entropic chain activities), and unfoldons (or conditionally disordered protein regions, which, in order to become functional or to make a protein active, have to undergo order-to-disorder transition) (Uversky, 2013c).

### Peculiarities of Conformational Behavior of IDPs/IDPRs

The free energy landscape of an extended IDP/IDPR is dramatically different from that of ordered globular protein or domain. In fact, if the energy landscape of an ordered protein has a funnel-like shape with a well-defined global energy minimum corresponding to unique protein structure (Radford, 2000; Jahn and Radford, 2005), the free energy landscape of an IDP can be described as a ‘hilly plateau,’ with hills on the plateau corresponding to the forbidden conformations (Uversky et al., 2008; Turoverov et al., 2010; Fisher and Stultz, 2011) and with numerous local energy minima. Such free energy landscape reflects the existence of numerous conformations that constitutes the dynamic conformational ensemble of an IDP. It also shows that such a protein that does not have stable conformation, possessing instead a highly frustrated nature (Uversky, 2013c). The shape of such energy landscape can be easily changed even by minimal changes in the local environment of an IDP. This contrasts high conformational stability of ordered proteins originated from their relatively robust funnel-like energy landscapes. Therefore, the ‘hilly plateau’-like energy landscape determines conformational plasticity of an IDP/IDPR and its ability to fold differently depending on the environmental conditions, where any (even rather subtle) changes in the IDP/IDPR environment might have very strong effects on the protein/region structure leading to the formation of very different structures (Uversky, 2013c).

Because of the IDPs/IDPRs are characterized by highly biased amino acid compositions, their conformational behavior is noticeably different from that of ordered proteins and domains. In fact, proteins with extended disorder (which are typically depleted in hydrophobic residues, but enriched in charged and polar residues) were shown to partially fold at increase in temperature, which is in striking opposition to globular proteins and domains that typically unfold at heating. This structure-forming effect of elevated temperature was attributed to the temperature-driven increase in the strength of the hydrophobic interactions that leads to a stronger hydrophobic attraction at higher temperatures (Uversky, 2002b). Similarly, a decrease (or increase) in pH was shown to induces partial folding of many extendedly disordered proteins characterized by high net charges at neutral pH. This is because at extreme pH, the net charge of such proteins decreases, causing the decrease in their charge-charge intramolecular repulsion. This permits formation of a partially folded conformation due to the hydrophobicity-driven collapse of polypeptide chain (Uversky, 2002b).

Intrinsically disordered proteins/IDPRs are characterized by highly dynamic and heterogeneous structures that are extremely sensitive to changes in the environment and that determine unique functional repertoire of disordered proteins. Since PTMs do alter local physical and chemical properties of proteins, changing their charge, flexibility, and hydrophobicity, such modifications serve as crucial regulators of IDP/IDPR functions and structures (Uversky, 2013b). Therefore, sections below, in addition to the introduction of the natural PTM variability, provide an outline of the roles that PTMs play in the regulation of IDPs/IDPRs.

## Posttranslational Modifications

As it follows from their definitions, PTMs are chemical changes affecting proteins after their biosynthesis, with almost every protein being potentially able to undergo PTMs. Changes induced by PTMs to protein structure are many. Various chemical groups, carbohydrates, lipids, and even entire proteins or nucleic acids can be covalently added to amino acid side chains. Proteins can also undergo enzymatic cleavage of peptide bonds or removal of various chemical groups. Since different PTMs can differently affect physicochemical properties of a protein (Mann and Jensen, 2003), different modifications can graft different functions to the same protein (Jungblut et al., 2008). Although natural variability of PTMs is very broad, these modifications are typically very specific. Many PTMs are catalyzed by special enzymes that recognize particular motifs in target sequences of specific proteins. Some PTMs (e.g., phosphorylation, acetylation, glycosylation, lipidation, methylation, and nitration) are readily reversible due to the concert action of modifying and demodifying enzymes. Such and interplay between the conjugating and deconjugating enzymes represents an economical and rapid way of the controlling the protein function. Furthermore, although mutations (which represent another means of changing the chemical properties of a polypeptide chain) can only occur once per position, different forms of PTMs may happen in tandem (Khoury et al., 2011).

### Diversity of PTMs and Their Classifications

Posttranslational modifications represent an important means for the increase in the variability and diversity of protein structures and functions via extension of the range of structures and physico-chemical properties of amino acids (Walsh et al., 2005). It was pointed out that although there are 20 primary amino acids typically utilized in protein biosynthesis, in reality, because of various PTMs, proteins might contain more than 140 chemically different residues. To this end, large portion of the genomes of higher eukaryotes (as much as 5%) represents genes that encode PTM-related enzymes. Altogether, as many as 300 PTMs are known to occur physiologically in proteins (Witze et al., 2007).

Posttranslational modifications are abundantly present in nature. For example, between at least one-fifth (Khoury et al., 2011) and a half of proteins are expected to be glycosylated (Apweiler et al., 1999). The most common PTMs are covalent removal or addition of various groups, formation of disulfide bonds, and specific cleavage of protein precursors (Baumann and Meri, 2004). According to the PTM Curator, most often proteins undergo phosphorylation, acetylation, N-linked glycosylation, amidation, hydroxylation, methylation, O-linked glycosylation, ubiquitylation, attachment of pyrrolidone carboxylic acid or gamma-carboxyglutamic acid, sulfation, sumoylation, palmitoylation, C-linked glycosylation, ADP-ribosylation, myristoylation, farnesylation, nitration, formylation, geranyl-geranylation, deamidation, *S*-nitrosylation, citrullination, *S*-diacylglycerol cysteine modification, GPI anchoring, bromination, and FAD addition (Khoury et al., 2011). Diversity of PTMs found in proteins is illustrated by **Table 1**, which not only shows that due to the various PTMs, side chains of all natural amino acids can be chemically diversified, but also emphasizes that each amino acid residue can be affected by many different PTMs. However, although all amino acid residues can be modified, most commonly PTMs affect side chains that can act as either strong (C, D, E, H, K, M, R, S, T, and Y) or weak (N and Q) nucleophiles (Walsh et al., 2005).

**Table 1:** Variability of posttranslational modifications of side chains in proteins.

Residue	Reaction	Example of a protein with indicated PTM
Alanine (Ala, A)	*N*-acetylation	N-alpha-acetyltransferase
	Amidation	Pantothenate synthetase
	*N*-methylation	Ribosomal proteins
Arginine (Arg, R)	*N*-methylation	Histones
	*N*-ADP-ribosylation	GSa
	Citrullination/Deimination	Argininosuccinate synthase
	*N*-acetylation	N-alpha-acetyltransferase
	Amidation	Tachykinins
	Dihydroxylation	Steilins
	Hydroxylation	Carbon monoxide dehydrogenase large
	Phosphorylation	chain
	*N*-glycosylation	Histones
		*N*-glycoproteins
Asparagine (Asn, N)	*N*-glycosylation	*N*-glycoproteins
	*N*-ADP-ribosylation	eEF-2
	Protein splicingDeamidation	Intein excision stepIsomerization to isoaspartate and
	Amidation	aspartate
	Hydroxylation	FMRFamide-related peptidesHypoxia-inducible factor 1-alpha
Aspartic acid (Asp, D)	Phosphorylation	Protein tyrosine phosphatases; response
	Isomerization to isoaspartate	regulators in two component systems
	Deamidation	Protein-L-isoaspartate *O*-
	*N*-acetylation	methyltransferase
	Beta-methylthiolation	Beta-casein
	Hydroxylation	N-alpha-acetyltransferase
	*Cis*-14-hydroxy-10,13-dioxo-	Ribosomal proteins
	7-heptadecenoic acid aspartate	3-hydroxyaspartate alsolase
	ester	Non-specific lipid transfer proteins
Cysteine (Cys, C)	*S*-hydroxylation (S-OH)	Sulfenate intermediates; peroxiredoxins
	Disulfide bond formation	Protein in oxidizing environments
	Phosphorylation	PTPases
	*S*-acylation	Ras
	*S*-prenylation	Ras
	Protein splicing	Intein excisions
	*N*-acetylation	N-alpha-acetyltransferase
	*N*-ADP-ribosylation	Glyceraldehyde-3-phosphate
	Amidation	dehydrogenase
	*S*-archaeol cysteine	Cystein synthase A
	Cysteine sulfinic acid (-SO_2_H)	Halocyanin
	Methylation	Cysteine sulfinic acid decarboxylase
	*N*-myristoylation	Crystallins
	Nitrosylation	Genome polyproteins of several viruses
	*N*-palmitoylation	Thioredoxins
	*S*-palmitoylation*S*-glutathionylation	Small cystein-rich outer membrane protein OmcA
	*S*-glutathionylation	Myelin proteolipid proteins
		Redox regulation *via* reversible glutathionylation
Glutamic acid (Glu, E)	Methylation	Chemotaxis receptor proteins
	Carboxylation	Gla residues in blood coagulation
	Polyglycination	Tubulin
	Polyglutamylation	Tubulin
	*N*-acetylation	N-alpha-acetyltransferase
	Poly *N*-ADP-ribosylation	Poly [ADP-ribose] polymerase 1
	Amidation	Buccalin
	Deamidation followed by methylation	Methyl-accepting chemotaxis proteins
Glutamine (Gln, Q)	Transglutamination	Protein cross-linking
	Deamidation	Myelin basic protein
	Amidation	FMRFamide-related peptides
	N-methylation	Ribosomal proteins
Glycine (Gly, G)	*C*-hydroxylation	C-terminal amide formation
	*N*-acetylation	N-alpha-acetyltransferase
	Amidation	Glycine oxidase
	Cholesterol glycine ester	Hedgehog proteins
	*N*-myristoylation	Protein Nef
Histidine (His, H)	Phosphorylation	Sensor protein kinases in two-component regulatory systems
	Aminocarboxypropylation	Diphthamide formation
	*N*-methylation	Methyl CoM reductase
	Amidation	VIP peptides
	Bromination	Sperm-activated peptide SAP-b
	Methylation	Actin
Isoleucine (Ile, I)	Amidation	FMRFamide neuropeptides
	*N*-methylation	Fimbial protein
Leucine (Leu, L)	Amidation	Myomodulin neuropeptides
	*N*-methylation	Major structural subunit of bundle-forming pilus
Lysine (Lys, K)	*N*-methylation	Histone methylation
	*N*-acylation by acetyl, biotinyl,	Histone acetylation; swinging-arm
	lipoyl, ubiquityl groups	prosthetic groups; ubiquitin; SUMO (small ubiquitin-like modifier) tagging of
	*C*-hydroxylation	proteins
	*O*-glycosylation	Collagen maturation
	*N*-acetylation	Adiponectin; *O*-glycoproteins
	Allysine	N-alpha-acetyltransferase
	Amidation	Elastin and collagen; lysyl oxidase
	N6-1-carboxyethylation	
	Dihydroxylation	Histone-lysine N-methyltransferase
	Hydroxylation	EHMT1
	*N*-myristoylation	Carbonyl reductases
	*N*-palmitoylation	Steilins
	Trimethylation	Collagens
		Tumor necrosis factors
		Serine palmitoyltransferases
		Myosins
Methionine (Met, M)	Oxidation to methionine	Methionine sulfoxide reductase
	sulfoxide	Catalase
	Oxidation to methionine	Unstable hemoglobin, Hb Bristol
	sulfone	[p67(E11) Val-Met]
	Silent modification	N-alpha-acetyltransferase
	(conversion to aspartic acid)	MIP-related peptides
	*N*-acetylation	Ribosomal proteins
	Amidation	
	*N*-methylation	
Phenylalanine (Phe, F)	Amidation	FMRFamide neuropeptides
	Hydroxylation	Adhesive plaque matrix proteins
	*N*-methylation	ComG operon proteins
Proline (Pro, P)	*C*-hydroxylation	Collagen; HIF-1a
	Dihydroxylation	Virotoxin
	*N*-acetylation	N-alpha-acetyltransferase
	Amidation	Prothyroliberin
	*N*-methylation	*N*-methylproline demethylase
Serine (Ser, S)	Phosphorylation	Protein serine kinases and phosphatases
	O-glycosylation	Notch *O*-glycosylation
	Phosphopantetheinylation	Fatty acid synthase
	Autocleavages	Pyruvamidyl enzyme formation
	*N*-acetylation	N-alpha-acetyltransferase
	*O*-acetylation	*O*-acetylserine (thiol) liase
	*N*-ADP-ribosylation	Ras-related protein Rap-1b
	Amidation	Kallikrein-8
	*N*-decanoylation	Ghrelin
	*O*-octanoylation	Appetite-regulating hormons; ghrelin
	*O*-palmitoylation	Myelin proteolipid protein
	Sulfation	Retrograde protein of 51 kDa
Threonine (Thr, T)	Phosphorylation	Protein threonine kinases/phosphatises
	*O*-glycosylation	*O*-glycoproteins
	*N*-acetylation	N-alpha-acetyltransferase
	Amidation	Aurora kinase A
	*N*-decanoylation	Ghrelin
	*O*-octanoylation*O*-palmitoylation	GhrelinMyelin proteolipid protein
	Sulfation	Cathepsin
	*O*-acetylation	Inhibitor of nuclear factor kappa-B kinase subunit alpha
Tryptophan (Trp, W)	*C*-mannosylation	Plasma-membrane proteins
	Amidation	Neuropeptide-like proteins
	Bromination	Mu-conotoxins
	C-linked glycosylation	Properdin
	Hydroxylation	Alpha-ketoglutarate-dependent taurine dioxygenase
Tyrosine (Tyr, Y)	Phosphorylation	Tyrosine kinases/phosphatases
	Sulfation	CCR5 receptor maturation
	*ortho*-nitration	Inflammatory responses
	TOPA quinine	Amine oxidase maturation
	*N*-Acetylation	N-alpha-acetyltransferase
	Amidation	FMRFamide-related neuropeptides
	*N*-methylation	General secretion pathway protein I
	*O*-glycosylation	S-layer protein SpaA
Valine (Val, C)	*N*-Acetylation	N-alpha-acetyltransferase
	Amidation	MIP-related peptides
	Hydroxylation	Conophans

All sides of protein life in a cell are affected by PTMs, which can regulate protein folding, target proteins to specific subcellular compartments, control interaction of proteins with their partners, modulate catalytic activity, or affect signaling and recognition functions (Mann and Jensen, 2003; Deribe et al., 2010). Functions of many proteins rely on multiple different PTMs, and modified sites in such multi-PTMed proteins are utilized individually for mediation of specific protein activities or used together for controlling molecular interactions and for modulation of the overall protein activity and stability (Yang, 2005). An illustrative example of such multi-PTMed proteins is given by histones, different stages of action of which require acetylation, ADP-ribosyation, methylation, phosphorylation, SUMOylation and ubiquitylation (Peng et al., 2012). The N-terminal tails of core histones protruding from the nucleosome core and needed to mediate chromatin compaction (Arya and Schlick, 2009) are known to contain an astonishing number of PTM sites (Shliaha et al., 2017). Furthermore, over 30 histone modifications have been also identified in the core domains of these proteins (Mersfelder and Parthun, 2006).

Because of their variability, PTMs can be grouped and classified on multiple ways. For example, depending on the stages of the protein life at which they were introduced, PTMs can be ‘early’ or ‘late’ and can give very different outputs. In fact, some proteins are known to be modified shortly after completion of their biosynthesis and before the final steps of their folding. The corresponding ‘early’ PTMs can influence the efficiency of protein folding, or affect protein conformational stability, or direct the nascent protein to distinct cellular compartments thereby defining its cellular fate (Tokmakov et al., 2012). The ‘late’ PTMs occur after the completion of protein folding and localization. They can activate, deactivate, or modulate the biological activity of target proteins (Ngounou Wetie et al., 2014). PTMs can also be classified based on the new modification-enabled functionality, such as gain of the catalytic function (e.g., resulting from phosphopantetheinylation, biotinylation, or lipoylation), membrane localization (e.g., caused by glypiation, geranylgeranylation, myristoylation, palmitoylation, prenylation, etc.), and proteolytic destruction by proteasomes (via ubiquitylation).

Another classification of PTMs is based on the underlying molecular mechanisms, such as functional group addition (e.g., phosphorylation, acylation, glycosylation, etc.), covalent conjugation of peptides and small proteins to the main polypeptide chain (e.g., ISGylation, neddylation, SUMOylation, ubiquitination, etc.), change in the physico-chemical properties of amino acids (citrullination, deamidation, deimidation, oxidation, etc.), and proteolytic cleavage (Xie et al., 2007a). PTMs can also be classified based on the description of the fragment of coenzyme or cosubstrate attached to the modified protein and the chemical nature of the protein modification, e.g., ATP-dependent phosphorylation, acetyl CoA dependent acetylation, NAD-dependent ADP ribosylation, CoASH-dependent phosphopantetheinylation, phosphoadenosinephosphosulfate (PAPS)-dependent sulfurylation, and *S*-adenosylmethionine (SAM)-dependent methylation (Walsh et al., 2005).

## Posttranslational Modifications and Intrinsic Disorder

Already in early IDP-related studies, it has been pointed out that many PTM sites are frequently associated with IDPRs (Dunker et al., 2002a). Therefore, in addition to the aforementioned classifications of PTMs based on the modulation molecular mechanisms or modulation-enabled functions, PTMs can also be grouped according to the conformational requirements of the potential modification sites. This classification generates two major PTM groups, where modifications are associated either with structured regions or with IDPRs (Xie et al., 2007a). **Tables 2**, **3** list some of the PTMs associated with IDPs/IDPRs and ordered proteins and domains, respectively (Xie et al., 2007a). Curiously, PTMs targeting ordered regions, such as covalent attachment of quinones and organic radicals, formylation, oxidation, and protein splicing introduce new chemical moieties needed for novel catalytic functions, or changing of the existing enzymatic activity, or modulation of the protein conformational stability. On the other hand, PTMs preferentially targeting IDPRs, such as phosphorylation, acetylation, and methylation, are typically used for modifications of the regulatory and signaling regions in target proteins that are engaged in specific but weak interactions with their partners. For example, p53- and p14-ARF-binding regions of the Mdm2 contain the majority of the phosphorylation sites of this protein. Similarly, the phosphorylation of PEST motifs of many proteins controls their ubiquitin-mediated degradation. The biological activities of various proteins involved in signal transduction are modulated by phosphorylation. Furthermore, this PTM can alter gene expression by modulating the binding affinity of transcription factors to their coactivators and DNA, and also can affect cell growth and differentiation (Zor et al., 2002).

**Table 2:** Top 20 of the PTM-related keywords strongly correlated with predicted disorder.

Keywords	Number of proteins	Number of families
Phosphorylation	10893	1651
Cleavage on pair of basic residues	867	189
Amidation	833	165
Ubl conjugation	805	152
Sulfation	245	47
Prenylation	722	41
Myristate	681	71
Lipoprotein	4335	624
Gamma-carboxyglutamic acid	106	14
Proteoglycan	189	27
Glycoprotein	16207	1941
Pyrrolidone carboxylic acid	791	196
Methylation	1417	99
D-amino acid	29	12
Heparan sulfate	48	10
Covalent protein-DNA linkage	26	8
Hydroxylation	334	47
GPI-anchor	590	146
Palmitate	2354	364
ADP-ribosylation	150	11

**Table 3:** All PTM-related keywords strongly correlated with predicted order.

Keywords	Number of proteins	Number of families
Quinine	449	17
Covalent protein-RNA linkage	108	4
Organic radical	54	3
TPQ	22	2
Zymogen	1680	120
PQQ	26	7
Formylation	57	27
Protein splicing	84	30
Autocatalytic cleavage	325	51
Oxidation	32	13

### Some PTMs Are Preferentially Found in IDPRs, Why?

Mechanistically, the prevalence of intrinsic disorder in the display sites of proteins targeted for certain PTMs is defined by the fact that structural pliability associated with high conformational dynamics of potential modification sites is crucial for the efficient action of a modifying enzyme on a multitude of different target proteins. In other words, such association between PTMs and IDPRs represents a solution for the apparent conundrum, where the modifying enzymes have to act following the ‘one-lock-many-keys’ scenario instead of the classical ‘lock-and-key’ mode of the enzyme action. The scale of the problem with too many ‘keys’ for a given ‘lock’ is given by kinases and phosphatases utilized in protein phosphorylation-dephosphorylation cycles. Protein kinases and phosphatases are among the largest gene families in eukaryotes [e.g., yeast and mouse genomes encode 119 and 540 kinases, respectively, and there are ∼520 (1019) kinase- and 150 (300) phosphatase-coding genes in human (or *Arabidopsis thaliana*) kinome]. However, in any given proteome, the number of proteins undergoing reversible phosphorylation is noticeably larger than the number of kinases and phosphatases. In fact, phosphorylation is expected to affect functions of at least one-third of eukaryotic proteins (Marks, 1996). In humans, more than two-thirds of the 21,000 proteins were shown to be phosphorylated, and it is likely that more than 90% are actually subjected to this type of PTM (Ardito et al., 2017). Based on the very conservative estimate of the penetrance of phosphorylation in human proteome (66.7%), each human kinase act on 27 target proteins, whereas each human phosphatase can dephosphorylate 93 clients. These numbers increase to 36 and 126 clients for each human kinase and phosphatase, respectively, if one would use the less conservative estimate of the prevalence of phosphorylation in human proteome (90%). However, the reality is even more complex, since many proteins are known to have multiple phosphorylation sites. Similar situation is observed for glycosylation (the covalent addition of carbohydrate moieties to specific amino acids), since almost half of all proteins typically expressed in a cell undergo this modification. Similarly, although the vast majority of human proteins end their lives via proteasomal degradation and although functionality and cellular localization of many proteins are regulated by ubiquitination–deubiquitination sites, the human genome codes for ∼600 E3 ubiquitinating ligases and 80 deubiquitinases (Komander et al., 2009).

Therefore, for each ‘lock’-containing enzyme (kinase, phosphatase, or any other modifying enzyme that targets multiple unrelated proteins) there are numerous ‘keys’ (modification sites of protein targets). This observation raises an important question on what allows these modifying enzymes to be engaged in the ‘one-lock-many-keys’ activity. An obvious solution is in relaxing classical ‘lock-and-key’ model by allowing structural flexibility. In fact, the problem is easily solved if instead of a ‘rigid key’ a modifiable protein is using a ‘flexible lock pick.’ In line with these consideration is a well-known fact that substrates are typically bound to the kinase rather weakly, despite the high specificity of the phosphorylation process. Such combination of low affinity and high specificity is a characteristic feature of signaling interactions (Bossemeyer et al., 1993; McDonald and Thornton, 1994; Hubbard, 1997; Lowe et al., 1997; Narayana et al., 1997; ter Haar et al., 2001), which are commonly based on intrinsic disorder (Dunker et al., 2001; Dunker et al., 2002a, 2005; Uversky et al., 2005). Often, such low affinity – high specificity protein-protein interactions rely on the coupled binding and folding of at least on of the partners. The corresponding disorder-to-order transition is characterized by a positive free energy change that reduces the magnitude of the negative free energy change associated with the interactions and defines the low binding affinity (Schulz, 1979).

**FIGURE 4:**
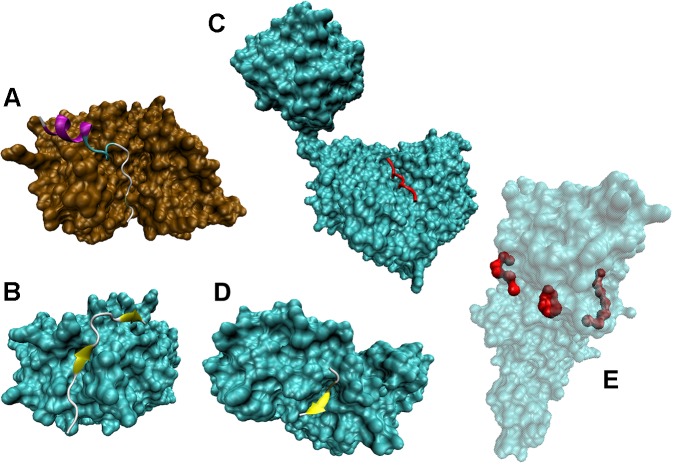
Disordered nature of the PTM sites in target proteins. **(A)** Crystal structure of a complex between a 20-amino acid peptide derived from the heat stable protein kinase inhibitor (PKIα) and the catalytic subunit of cyclic AMP-dependent protein kinase (cAPK) (PDB ID: 2CPK) (Knighton et al., 1991). **(B)** Crystal structure of a complex between a 23 amino acid cell-permeable peptide and a protein Ser/Thr phosphatase-1 (PDB ID: 4G9J) (Chatterjee J. et al., 2012). **(C)** Crystal structure of a complex between polypeptide *N*-acetylgalactosaminyltransferase 2 and a 13 amino acid peptide EA2 (PDB ID: 2FFU) (Abraham and Podell, 1981). **(D)** Crystal structure of a complex between human mitochondrial NAD-dependent deacetylase sirtuin-3 and a 12 amino acid peptide derived from mitochondrial acetyl-coenzyme A synthetase 2-like (PDB ID: 3GLT) (Jin et al., 2009). **(E)** Crystal structure of a complex between a protein arginine *N*-methyltransferase 1 and a 19 amino acid substrate peptide (PDB ID: 1OR8) (Zhang and Cheng, 2003).

**Figure 4** further illustrates the mostly disordered nature of regions containing various PTM sites. Crystal structures of complexes between several modifying enzymes, such as kinase, phosphatase, glycosyltransferase, deacetylase, and methyltransferase, and peptides derived from their corresponding target proteins are shown (see **Figures 4A–E**, respectively). In all these cases, despite the obvious differences between proteins and peptides, the mechanism of interaction, where an extended peptide is bound to the grove of an enzyme, is remarkably similar. **Figure 4A** shows a crystal structure a 20-amino acid peptide derived from the heat stable protein kinase inhibitor (PKIα) bound to the catalytic subunit of cyclic AMP-dependent protein kinase (cAPK) (PDB ID: 2CPK). In its bound from, the PKIα peptide is characterized by a highly extended conformation. Although this extended bound form is stabilized by a well-developed network of 36 H-bonds, only two of these bonds are intramolecular, with 16 H-bonds being formed with cAPK and remaining 18 H-bonds being formed with water. A very similar situation is observed for complexes between the protein Ser/Thr phosphatase-1 and a 23 amino acid cell-permeable peptide (PDB ID: 4G9J, **Figure 4B**), or the polypeptide *N*-acetylgalactosaminyltransferase 2 and the 13 amino acid peptide EA2 (PDB ID: 2FFU, **Figure 4C**), or human mitochondrial NAD-dependent deacetylase sirtuin-3 and a 12 amino acid peptide derived from the mitochondrial acetyl-coenzyme A synthetase 2-like (PDB ID: 3GLT, **Figure 4D**), or the protein arginine *N*-methyltransferase 1 and a 19 amino acid substrate peptide (PDB ID: 1OR8, **Figure 4E**).

Furthermore, systematic bioinformatics analyses of the peculiarities of the IDP/IDPR-located display sites targeted for PTMs and their adjacent regions showed that their sequence attributes (such as sequence complexity, charge, hydrophobicity, amino acid compositions, etc.) are very similar to those of IDPRs. For the first time, this observation was made for protein phosphorylation (Iakoucheva et al., 2004) and later similar trends were found for sites targeted for methylation (Daily et al., 2005), ubiquitination (Radivojac et al., 2010), *S*-palmitoylation (Reddy et al., 2017), as well as in protein regions that undergo multiple homologous or heterologous PTM events (Pejaver et al., 2014). This last observation is especially interesting, since it clearly indicates the importance of intrinsic disorder for the PTM-based regulation of proteins that occur not only through the individual effect of a given PTM at a single residue, but also through combined effects over multiple sites undergoing the same or different PTMs (Pejaver et al., 2014). These proteins affected by more than one PTM are commonly involved in transcriptional, posttranscriptional, and developmental processes contain multi-PTM or shared-PTM display sites that are characterized by preferences toward IDPRs exceeding those of the single-PTM sites (Pejaver et al., 2014). Furthermore, it was indicated that MoRFs possess significant preferences for PTM sites, particularly shared PTM sites, suggesting that PTMs play crucial roles in the modulation of this specific type of macromolecular recognition (Pejaver et al., 2014).

Also, the facts that >50% of all proteins are glycosylated (Apweiler et al., 1999; Ben-Dor et al., 2004), but only ∼5% of all PDB entries have attached glycan chains (Lutteke et al., 2004) indicate that glycosylated proteins are often disordered. In agreement with this hypothesis, an analysis of the complete proteomes of eight typical monocotyledonous and dicotyledonous plant species revealed that phosphorylation, acetylation, and *O*-glycosylation sites were preferentially located within the IDPRs of plant proteins (Kurotani et al., 2014). Similarly, a computational analysis of 20 algae proteomes revealed that phosphorylation, *O*-glycosylation, and ubiquitination sites, as well as PEST motifs [i.e., regions rich in proline, glutamic acid, serine, and threonine that serve as a signal for protein degradation (Rechsteiner and Rogers, 1996)] preferentially occurred in IDPRs (Kurotani and Sakurai, 2015).

Concluding, all these findings strongly suggest that many protein PTM sites are very commonly positioned within the IDPRs. Likely, this is because of the need of the corresponding modifying enzymes to work with a multitude of target sites in a wide variety of rather different protein targets (e.g., to utilize the ‘one-lock-many-keys’ mechanism). Disorder in flanking regions of such PTM sites provides a mean for a single modifying enzyme to bind and modify a wide variety of protein targets via a ‘flexible-lock-pick’ approach (Uversky, 2013b; Sirota et al., 2015).

### Structural and Functional Consequences of PTMs in IDPs/IDPRs

Because PTMs are associated with the addition of various chemical groups to target protein, they clearly represent one of the means of altering of the energy landscape of a protein, thereby leading to conformational changes. Curiously, there is no uniform response of a protein structure to PTMs. It was pointed out that the outputs of PTMs are very diverse, ranging from local stabilization or destabilization of transient secondary structure to global disorder-to-order transitions (Bah and Forman-Kay, 2016). PTMs can also drive global changes in the protein phase states, for example, driving transitions between intrinsically disordered and ordered states of a protein molecule or between the dispersed monomeric and phase-separated states (Bah and Forman-Kay, 2016).

Based on the analysis of the effects of phosphorylation on structural properties of 17 proteins with available structural information for their phosphorylated and non-phosphorylated forms it has been concluded that the types and extent of structural changes could be highly diverse, ranging from local to long-range structural changes, leading to both association and disassociation of protein complexes, and causing both order-to-disorder and disorder-to-order transitions (Johnson and Lewis, 2001). Extension of this analysis to all proteins for which structures corresponding to their modified and unmodified forms are known revealed that PTMs typically induce small but statistically significant conformational changes at both local and global levels (Xin and Radivojac, 2012). A few illustrative examples of how PTMs might affect structure and functionality of individual IDPs/IDPRs are given below.

A systematic structural analysis revealed that different PTMs (phosphorylation and acetylation) have a profound effect on the conformational preferences of the intrinsically disordered negative regulatory domain (NRD) of the p53 tumor suppressor, thereby regulating activity of this important protein (McDowell et al., 2013). Phosphorylation induces structural changes in the p65 subunit of NF-κ B, allowing subsequent p65 ubiquitination and interaction with transcriptional cofactors (Milanovic et al., 2014). Phosphorylation of the ETS domain transcription factor Elk-1 by extracellular signal-regulated kinase (ERK) modulates the interaction of Elk-1 with Mediator and histone acetyltransferases (Galbraith et al., 2013). Phosphorylation of the *Drosophila* transcription factor Hox at multiple sites regulates interactions of this protein with DNA and other proteins, and plays a role in transcription activation (Tan et al., 2002; Bondos et al., 2006; Liu et al., 2008, 2009). Comparison of the solution structures of the phosphorylated and non-phosphorylated forms of the E7 protein from HPV-16 revealed that phosphorylation has significant local effect, changing structural and dynamic properties of the 26–29 region located in the close proximity to the retinoblastoma tumor suppressor (pRb) binding LXCXE motif (residues 22–26), thereby affecting the interactability of this protein (Nogueira et al., 2017). Phosphorylation of a member of the group-3 LEA protein family, PM18 protein, did not affect global intrinsically disordered status of this protein, but had profound effects on the salt-tolerance-related functions of this soybean protein (Liu et al., 2017). Combined experimental and computational analysis of the effect of phosphorylation on the conformational preferences of the synaptotagmin 1 IDPR revealed that phosphorylation of Thr^112^ resulted in the disruption of a local disorder-to-order transition likely due to the induction of salt bridges unsuitable for helix formation (Fealey et al., 2018).

Progressive acetylation has a cumulative effect on the intrinsically disordered tail of the H4 core histone, leading to the decrease in its conformational heterogeneity, combined with the increase in helical propensity and hydrogen bond occupancies, as well as with the formation of spatially clustered lysines that could serve as recognition patches for interaction with proteins engaged in chromatin regulation (Winogradoff et al., 2015). The aforementioned cumulative effects of acetylation were shown to be associated with the reduction in the protein net charge and the increase in hydrophobicity caused by the addition of the acetyl groups (Winogradoff et al., 2015). Multiple combinatorial PTMs of the C-terminal domain of RNA Polymerase II was shown to coordinate transcription with mRNA processing as well as regulate multiple stages of transcription initiation (Yogesha et al., 2014). Activity of dehydrin/response ABA protein is likely to be regulated by multiple PTMs, such as acetylation, amidation, glycosylation, methylation, myristoylation, nitrosylation, *O*-linked β-*N*-acetylglucosamination, palmitoylation, phosphorylation, sumoylation, sulfation, and ubiquitination (Kalemba and Litkowiec, 2015). It was pointed out that human proteins with multiple PTM sites (Mtp-proteins) contain more IDPRs than proteins carrying no known PTM sites (Huang et al., 2014). These Mtp-proteins were shown to be significantly enriched in protein complexes, have more protein partners, and prefer to act as hubs/superhubs in protein–protein interaction (PPI) networks than the proteins carrying no known PTM sites (Huang et al., 2014).

### Intrinsic Disorder, PTMs, and Human Diseases

Various PTMs can control, modulate, and regulated functions of IDPs and IDPRs. Therefore, human diseases can be caused by aberrant PTMs. In agreement with this hypothesis, all major PTMs, such as acetylation, glycosylation, methylation, palmitoylation, phosphorylation, proteolytic degradation, and ubiquitination, can be altered in various human maladies, including cancer (Markiv et al., 2012), cardiovascular diseases, diabetes, and neurodegenerative diseases (Uversky, 2014). Systematic computational analysis revealed that ∼5% of the disease-associated mutations in human proteins may affect known PTM sites, with most of the 15 PTM types being found to be disrupted at levels higher than expected by chance (Li et al., 2010). Furthermore, the aforementioned Mtp-proteins were shown to be more prone to be involved in various human diseases than proteins carrying no known PTM sites (Huang et al., 2014).

Many malignancies [e.g., colorectal cancer (CRC) (Park and Lee, 2013)] are characterized by the abnormal glycosylation, which is commonly associated with the oncogenesis and cancer progression (Tuccillo et al., 2014). Many biomarkers used for diagnosis, prediction, and prognosis of various cancers are characterized by the aberrant N-linked glycosylation (Drake et al., 2006; Tian and Zhang, 2013; Hauselmann and Borsig, 2014). It was also shown that both gain and loss of phosphorylation target sites caused by the somatic mutations may play an active role in cancer pathogenesis (Radivojac et al., 2008). The distortion in the tightly controlled multiple modifications of the important nuclear IDPs, histones (Peng et al., 2012), is commonly found in malignancies (Campbell and Turner, 2013). For example, CRC is characterized by the abnormal acetylation and methylation of specific histone residues (Gargalionis et al., 2012), indicating the usefulness of the histone modification analysis for the diagnosis and prognosis in the CRC patients (Gezer and Holdenrieder, 2014). Alterations of the PTMs of lysine residues (such as methylation, acetylation, sumoylation, and ubiquitination) of proteins involved in DNA repair are frequently associated with genomic instability, which is the major cause of different diseases, especially cancers (Chatterjee S. et al., 2012). Normal and pathological activities of one of the high-mobility group transcription factors, the Sry-containing protein Sox2, are controlled by normal and pathological phosphorylation, acetylation, ubiquitination, methylation, and SUMOylation (Liu et al., 2013). Levels of a master transcriptional repressor REST/NRSF protein (RE-1 silencing transcription factor or neuron-restrictive silencer factor) that can serve as a tumor suppressor or oncogene are controlled by ubiquitination-deubiquitination cycles, with the abnormal upregulation of this protein being detected in glioblastoma, medulloblastoma, and neuroblastoma (Huang and Bao, 2012). Ectodomain shedding of syndecans, which is the proteolytic processing of cell-surface proteoglycans (PGs), is associated with the facilitation of cancer development. It also promotes cancer cell motility and invasion, thereby stimulating aggressiveness of various tumors (Theocharis et al., 2010).

In neurodegeneration, Huntington’s disease (HD) is characterized by aberrant acetylation, methylation, phosphorylation, polyamination, and ubiquitination of histones (Moumne et al., 2013). Furthermore, significant alterations in acetylation, palmitoylation, phosphorylation, proteolytic cleavage, sumoylation, and ubiquitination are reported for the HD causative protein, Huntingtin (Htt) (Ehrnhoefer et al., 2011). In Alzheimer’s disease (AD), levels and aggregation of the causative amyloid-β (Aβ) are affected by aberrant proteolytic cleavage of the amyloid precursor protein (APP). Pathogenesis of AD and other tauopathies is associated with altered sumoylation (Lee et al., 2013), abnormal hyperphosphorylation (Hernandez and Avila, 2007; Wang et al., 2013), and abnormal truncations of the microtubule-associated protein tau (Kovacech and Novak, 2010). The pathogenesis of frontotemporal lobar degeneration (FTLD) is associated with the aberrant phosphorylation of a RNA/DNA binding protein TDP-43 (TAR DNA binding protein 43) (Buratti and Baralle, 2008). In the transmissible spongiform encephalopathy (TSE) and other prion diseases, the infectious properties of the prion protein can be altered by changes in the glycosylation status of this protein (Cancellotti et al., 2013).

Acquired cardiac disorders, such as arrhythmias and heart failure, are associated with the aberrant functions of the voltage-gated sodium channel isoform 1.5 (NaV1.5) caused by its altered PTMs (Herren et al., 2013). The myofilament dysfunction in dilated cardiomyopathy (DCM) is associated with the aberrant phosphorylation of troponins I and T and myosin light chain, as well as with altered oxidation and glycation of sarcomeric proteins (LeWinter, 2005). Deregulated phosphorylation and glutathionylation of nitric oxide synthases NOS1 and NOS3 represent an important contributing factor in the pathogenesis of cardiac hypertrophy and failure, myocardial infarction, myocardial ischemia, reperfusion injury, and vascular disease (Carnicer et al., 2013).

In diabetes and hyperhomocysteinemia, the aberrant glycation of fibrinogen (Hammer et al., 1989; Henschen-Edman, 2001) is associated with the increased atherothrombotic risk (Hoffman, 2008). Also, prolonged increase in the *O*-GlcNAcylation and sustained increase in the *O*-GlcNAc (*O*-linked *N*-acetylglucosamine) levels are associated with the insulin resistance and glucose toxicity (McLarty et al., 2013). This is due to the distortions in the complex interplay between phosphorylation and *O*-GlcNAcylation (McLarty et al., 2013), since the rise in the GlcNAcylation levels can efficiently modulate the phosphate stoichiometry at most of the sites undergoing phosphorylation-dephosphorylation (Wang et al., 2008). Curiously, 381 proteins affected by these diabetes-distorted dual modifications (phosphorylation and *O*-GlcNAcylation) were shown to have a multitude of biological functions, acting as metabolic enzymes, cytoskeleton regulatory proteins, chaperones, kinases, RNA processing proteins, or transcription factors (Wang et al., 2008).

## Concluding Remarks

Intrinsic disorder represents a common property found in proteins engaged in recognition, regulation, and control of various signaling events and pathways. Interactions with biological binding partners cause partial or complete folding of many IDPs/IDPRs. Due to the multiple binding specificities and mechanisms, these proteins can be involved in one-to-many and many-to-one interactions. Normally, functions and abundance of IDPs/IDPRs are tightly controlled and modulated by various means, including a wide spectrum of PTMs. Alteration of the mechanisms controlling IDP/IDPR functionality, localization, and cellular levels can be detrimental, causing various maladies. Often, protein-based pathogenicity originates from aberrant PTMs and altered degradation of IDPs/IDPRs. It seems that PTMs represent very important cellular mechanisms that affect the abundance, cellular distribution, foldability, or functionality of IDPs/IDPRs, with altered PTMs being commonly associated with pathological transformations of these important cellular players.

## Author Contributions

VU conceived the idea. VU and AD collected and analyzed the literature data, and wrote and edited the manuscript.

## Conflict of Interest Statement

The authors declare that the research was conducted in the absence of any commercial or financial relationships that could be construed as a potential conflict of interest.
